# Interleukin-6 does not upregulate pro-inflammatory cytokine expression in an *ex vivo* model of giant cell arteritis

**DOI:** 10.1093/rap/rkz011

**Published:** 2019-05-06

**Authors:** Lorraine O’Neill, Jennifer McCormick, Wei Gao, Douglas J Veale, Geraldine M McCarthy, Conor C Murphy, Ursula Fearon, Eamonn S Molloy

**Affiliations:** 1 Centre for Arthritis and Rheumatic Diseases, St Vincent’s University Hospital, Dublin Academic Medical Centre, Royal College of Surgeons, Ireland; 2 Mater Misericordiae University Hospital, Dublin Academic Medical Centre, Royal College of Surgeons, Ireland; 3 Department of Ophthalmology, Royal Victoria Eye and Ear Hospital, Royal College of Surgeons, Ireland

**Keywords:** giant cell arteritis, interleukin-6, temporal artery culture, tocilizumab

## Abstract

**Objective:**

The aim of this study was to examine the pro-inflammatory effects of IL-6 in *ex vivo* temporal artery explant cultures.

**Methods:**

Patients meeting 1990 ACR classification criteria for GCA were prospectively recruited. Temporal artery biopsies were obtained and temporal artery explants cultured *ex vivo* with IL-6 (10–40 ng/ml) in the presence or absence of its soluble receptor (sIL-6R; 20 ng/ml) for 24 h. Explant supernatants were harvested after 24 h and assayed for IFN-γ, TNF-α, Serum amyloid A, IL-1β, IL-17, IL-8, angiotensin II and VEGF by ELISA. Myofibroblast outgrowths, cytoskeletal rearrangement and wound repair assays were performed.

**Results:**

IL-6 augmented production of VEGF, but not of any of the other pro-inflammatory mediators assayed. No differences were observed in the explants cultured in the presence or absence of the sIL-6R or between those with a positive (*n* = 11) or negative (*n* = 17) temporal artery biopsy. IL-6 did not enhance myofibroblast proliferation or migration. Western blot analysis confirmed signalling activation, with increased expression of pSTAT3 in response to IL-6+sIL-6R.

**Conclusion:**

IL-6 stimulation of temporal artery explants from patients with GCA neither increased expression of key pro-inflammatory mediators nor influenced myofibroblast proliferation or migration.

**Key messages**

- IL-6 may not be the key driver of vascular inflammation in GCA.
- The effect of IL-6 on vascular inflammation in GCA requires further study.

## Introduction

GCA is the most common primary systemic vasculitis in adults. Its prevalence is highest in Northern Europe, with annual incidence rates of 17 per 100 000 of the population >50 years of age [1]. The pathological hallmark of GCA is granulomatous inflammation of medium and large arteries. Arterial wall inflammation can ultimately result in complete occlusion of the involved vessel and consequent tissue ischaemia, with irreversible visual loss occurring in 4.4–20% of patients and stroke in 2–7% [2–5].

IL-6 is a pleiotropic cytokine with a broad range of biological functions and is produced by a wide variety of cell types in response to various stimuli. IL-6 contributes significantly to the acute phase response, with dysregulated production or signalling thought to contribute to many inflammatory and autoimmune conditions, including GCA [6, 7].

IL-6 exerts it effects by binding to its specific membrane-bound receptor (IL-6R) to form a complex with gp130, with subsequent activation of JAK/STAT signalling pathways. This process of activation via the membrane-bound IL-6R is known as classical signalling and is thought to contribute to the anti-inflammatory effects of IL-6. Although gp130 is ubiquitously expressed on all cell types, membrane-bound IL-6R is found on only certain cells, predominantly monocytes, macrophages, megakaryocytes, hepatocytes, B cells and certain T-cell subsets [8–10]; therefore, cells lacking the membrane-bound IL-6R are unresponsive to the direct effects of IL-6. To overcome this, all other cells not expressing membrane-bound receptor signal via trans-signalling, whereby IL-6 binds to the soluble form of the IL-6R (sIL-6R), which then can form a complex with gp130 on the cell surface.

IL-6 is postulated to play a role in the pathogenesis of GCA. Elevated serum levels of IL-6 have been found in patients with untreated PMR and GCA [11, 12]. GCA patients who have a relapsing disease course have higher circulating IL-6 levels than those achieving sustained remission [13, 14]. In addition, elevated circulating IL-6 is associated with increased glucocorticoid requirements, a longer duration of therapy and a stronger systemic inflammatory response [14, 15].

IL-6 is produced at the site of inflammation in GCA [15, 16] and is thought to derive from monocytes and macrophages, which are co-localized with activated T cells in the arterial wall [17]. Tissue expression of IL-6 is increased in those with a stronger systemic inflammatory response [15] and decreases post-treatment [13].

However, the direct effects of IL-6 as a potential driver of vascular inflammation in GCA have been unexplored to date. The aim of this study, therefore, was to examine the ability of IL-6 to induce pro-inflammatory mediators and to influence myofibroblast proliferation in an *ex vivo* temporal artery explant culture model.

## Methods

### Patient recruitment

Patients presenting with suspected GCA were recruited prospectively. The Research and Ethics Committees of all participating institutions approved this study. All patients provided informed consent. Standardized clinical and laboratory assessments were performed on all patients before temporal artery biopsy. One third of each biopsy was transferred to the research laboratory and *ex vivo* temporal artery whole tissue explant cultures were established, as previously described [16]. The remainder of the biopsy was sent to the routine pathology laboratory for histological diagnosis. Blood was obtained for isolation of peripheral blood mononuclear cells (PBMCs) and serum for measurement of circulating cytokines.

### Quantification of serum IL-6 and sIL-6R

Circulating IL-6 and the sIL-6R (R&D Systems, Minneapolis, USA) were quantified by ELISA. All ELISAs were performed in accordance with the instructions of the manufacturers. Absorbance was measured at 450 nm in a microtiter plate spectrophotometer (Dynatech MR4000, Alexandria, VA, USA).

### Isolation and culture of PBMCs

Blood obtained from GCA patients at their baseline assessment was drawn into heparin-containing tubes. It was subsequently diluted in a 1:1 ratio with Hanks balanced salt solution (Gibco BRL, Cheshire, UK) and underlaid with a Ficoll–metrizoate gradient (Lymphoprep; Nycomed UK Ltd, High Wycombe, UK) at a density of 1.077 g/ml. After centrifugation at 400*g* for 25 min, cells from the interface between the sample and lymphoprep were collected and washed twice in Hanks balanced salt solution by centrifugation.

PBMCs were seeded at a cell density of 500 000 cells/250 µl in full DMEM containing recombinant human IL-6 (rhIL-6; 20 ng/ml) for 24 h. Cultured supernatants were harvested and quantified by ELISA.

### The effect of IL-6 on cytokine and angiogenic factors using temporal artery tissue explant cultures *ex vivo*

To examine directly the effect of IL-6 on pro-inflammatory cytokines and growth factor expression in GCA, temporal artery explant tissues were sectioned into 96-well plates (Falcon, Franklin Lakes, NJ, USA) in full DMEM and incubated with rIL-6 (10–40 ng/ml) with or without sIL-6R (20 ng/ml) for 24 h at 37°C in air supplemented with 5% CO_2_. Supernatants were harvested and assayed for IFN-γ, TNF-α, serum amyloid A (SAA), IL-1β, IL-17, IL-8, angiotensin II and VEGF by ELISA.

### RA synovial tissue explant culture

Given that IL-6 has been shown to promote IL-8 production in RA synoviocytes [18], as a control, RA synovial tissue was obtained at the time of arthroscopy and established as whole tissue synovial explants as previously described [19], cultured with rhIL-6 in identical conditions to the temporal artery explants. IL-8 was measured in the cultured supernatants by ELISA (R&D Systems), to establish whether tissue-specific differences in response could be observed.

### The effect of IL-6 inhibition on basal cytokine release

We have previously reported spontaneous release of IL-6 and IL-8 from GCA temporal artery explants after 24 h in culture [16]. To address whether IL-6 inhibition has an effect on blocking basal cytokine release, explants were cultured in the presence of anti-human IL-6 (10 μg/ml; R&D Systems) or an IgG control (10 μg/ml; R&D Systems) for 24 h, and IL-8 levels in the harvested supernatants were measured by ELISA.

### Isolation and culture of myofibroblasts from temporal artery biopsies

To examine the effect of IL-6 on migration/invasion in GCA, myofibroblast outgrowth experiments were performed. Matrigel (50 μl) was plated in 96-well culture plates and allowed to polymerize at 37°C in air supplemented with 5% CO_2_. Temporal artery explants were carefully embedded in the Matrigel. Explants were stimulated with IL-6 in full DMEM (10–40 ng/ml; R&D Systems) over a time course of 1–21 days. Supernatants were collected every 3 days and replenished with fresh media and experimental agents. Once the outgrowths cultured in basal conditions as described above were confluent, myofibroblasts were trypsinized (Clonetics, San Diego, CA), transferred to uncoated six-well plates, cultured in full DMEM and grown to confluence. Positive immunofluorescence staining for F-actin and vimentin was confirmed in the cultured myofibroblasts. Myofibroblasts were seeded into eight-well chamber slides, stimulated for 24 h with IL-6 or A-SAA and subsequently stained for F-actin.

Chamber slides were rinsed in PBS and fixed in 3.7% paraformaldehyde in PBS for 20 min. After rinsing in PBS, cells were permeabilized with 0.1% Triton X-100 for 3 min and then rinsed three times in PBS. To visualize F-actin, slides were stained for 40 min at room temperature with fluorescein isothiocyanate–phalloidin (Molecular Probes). Nuclei were counterstained with 4′,6-diamidino-2-phenylindole dihydrochloride (Sigma, Watford). Stained cells were visualized with a Leitz DM40 microscope and images captured using an AxioCam system with AxioVision v.3.0.6 software (Carl Zeiss, Cambridge, UK). Staining of individual cytoskeletal filaments running in parallel to the long axis of the cell and pseudopodal projections, both typical of myofibroblasts, were clearly seen.

### Wound repair assay

Myofibroblasts were seeded into 96-well plates. The monolayers were then wounded with a sterile pipette tip and treated with IL-6 (20 ng/ml) and A-SAA (1 μg/ml; InvivoGen, Toulouse, France) in full DMEM. Myofibroblast migration across the wound margins from 24 h was assessed and photographed using a phase-contrast microscope (Nikon TMS microscope linked to a Canon S70 camera).

### Western blot analysis

To investigate the effect of IL-6 with or without sIL-6R on signal transduction in the temporal artery explants, pSTAT3 expression was assessed by western blot. After culture, temporal artery biopies were snap frozen and subsequently powdered using a Mikro-dismembrator U (B Braun Biotech, Melsungen, Germany). Powdered biopsy specimens were lysed, and protein was extracted in ice-cold radioimmunoprecipitation assay buffer (Sigma-Aldrich, Dorset) containing 10 μg/ml phosphatase inhibitor cocktail and 10 μg/ml protease inhibitor cocktail (Sigma-Aldrich). The protein concentration was measured using a bicinchoninic acid assay (Pierce). Protein lysates were then resolved by SDS–PAGE (10% resolving, 5% stacking) before being transferred onto nitrocellulose membranes. Membranes were blocked for 1 h at room temperature in wash buffer containing 5% non-fat dry milk, with gentle agitation. After two 10-min washes in wash buffer, membranes were incubated with pSTAT3 (Cell Signaling Technology, London; catalogue no. #9145, 1:500 dilution) diluted in PBS containing 0.05% Tween 20 and 5% non-fat dry milk at 4°C overnight with gentle agitation. We used β-actin (Sigma; catalogue no. A5441, 1:5000 dilution) as a loading control. After two additional 10-min washes, membranes were incubated in horseradish peroxidase-conjugated anti-rabbit IgG (1:1000 dilution for pSTAT3) or anti-mouse IgG (1:10 000 dilution for β-actin) for 2 h. After three final 10-min washes, the Enhanced chemiluminescence (ECL) detection reagent was placed on the membranes for 5 min before they were exposed to Hyperfilm ECL (Pierce ECL, ThermoFisher, Waltham, MA, USA). The signal intensity of the appropriate bands on the autoradiogram was calculated using the autochemi system UVP bioimaging AutoChemi System (Analytikjena, Upland, CA, USA).

### Statistical analysis

Graphpad Prism v.6.0d (GraphPad Software, San Diego, CA, USA) was used for statistical analysis. For non-parametric data, the Wilcoxon signed rank test for related samples and Mann–Whitney *U*-test for non-paired samples were used. Student’s *t*-tests were used to analyse parametric data. A vlaue of *P *<* *0.05 was deemed statistically significant. Results are expressed as the mean (s.e.m.).

## Results

Temporal artery biopsies obtained from 28 patients with GCA are included in this analysis. The mean age of the cohort at diagnosis was 73 ±11 years, and the majority (75%) were female. Key demographic, clinical and laboratory features of the cohort are outlined in Supplementary Table S1, available at *Rheumatology Advances in Practice* online. All patients were on prednisolone at the time of temporal artery biopsy, and all biopsies were performed within 10 days of starting treatment (mean 6.6 days, range 1–10 days).

### Serum levels of IL-6 and sIL-6R

**Fig. 1:**
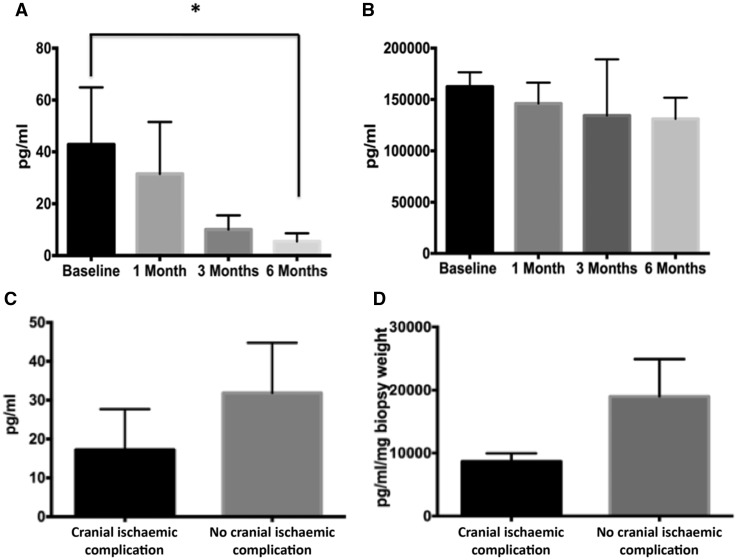
Serum IL-6 in GCA Serum IL-6 (**A**) and soluble IL-6 receptor (sIL-6R; **B**) levels in patients with GCA. The serum concentration was measured (in picograms per millilitre) by ELISA. The data represent the mean (s.e.m.). *P ≤ 0.05, significantly different. Circulating levels (**C**) and spontaneous release in temporal artery explant culture (**D**) of IL-6 in patients presenting with a cranial ischaemic complication of GCA (*n* = 2) and those without (*n* = 26).

Although circulating serum IL-6 levels in patients with GCA were elevated at baseline, levels were highly variable between patients, with a mean baseline value of 42.81 ± 22.08 pg/ml. Serum IL-6 levels declined significantly after glucocorticoid treatment, with mean values of 5.45 ± 3.12 pg/ml detectable at 6 months (Fig. 1A). Significant levels of the sIL-6 receptor were detected at baseline, and they were unaffected by glucocorticoid treatment, with mean values at presentation of 157 700  ± 15 656 pg/ml and after 3 months of glucocorticoid therapy of 146 211 ± 22 069 pg/ml (Fig. 1B). Patients presenting with a cranial ischaemic event had lower mean baseline circulating IL-6 levels [17.23 ± 10.45 *vs* 31.83 ± 12.95 pg/ml; Fig. 1C]. This effect was paralled in temporal artery explants, where a decrease in spontaneous release of IL-6 from temporal artery explants was seen (8660 *vs* 18 920 pg/ml/mg biopsy weight; Fig. 1D). However, only 2 of the 28 patients included in this analysis presented with an ischaemic event.

### The effect of rhIL-6 on pro-inflammatory mediators from GCA PBMCs and temporal artery explant cultures

**Fig. 2:**
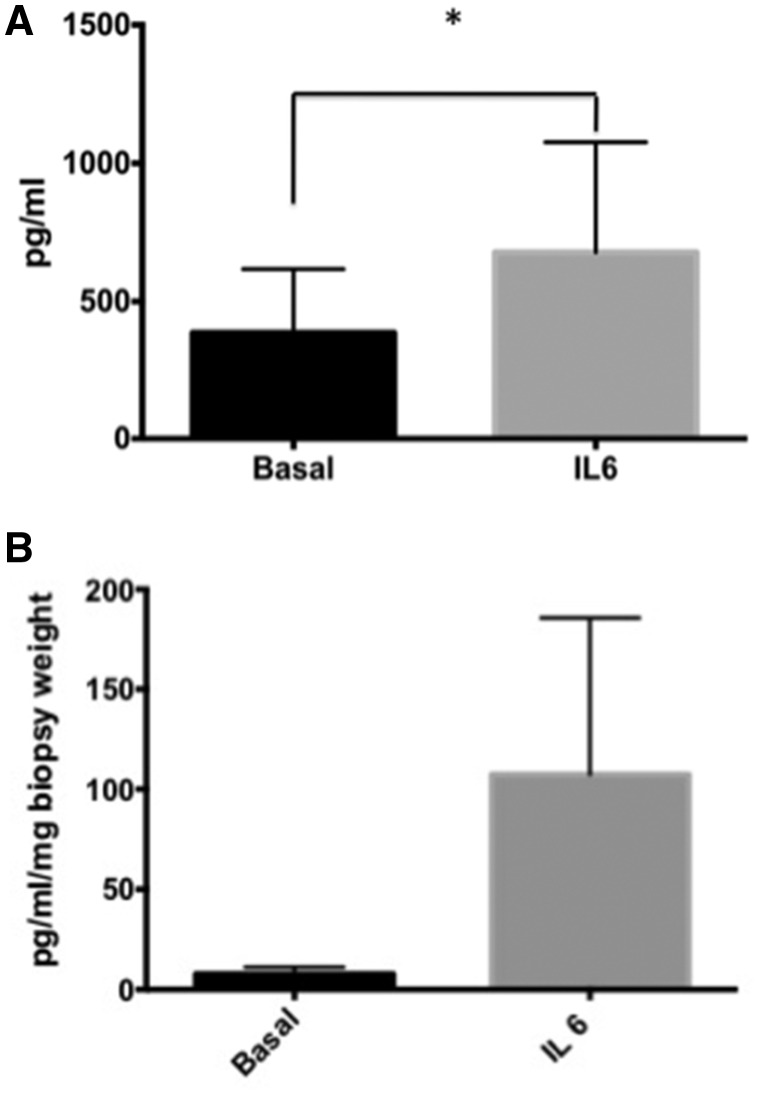
Increased IL-8 and VEGF expression following stimulation with IL-6 (**A**) IL-6 significantly induced expression of IL-8 in peripheral blood mononuclear cell (PBMC) cultures from patients with GCA. The *y*-axis shows the concentration of IL-8 in supernatants from PBMC cultures as measured by ELISA. (**B**) In temporal artery explant cultures, IL-6 in isolation was sufficient to induce expression of VEGF (*n* = 28), consistent with the known ability of IL-6 to promote angiogenesis. The *y*-axis shows the concentration of VEGF in supernatants from temporal artery explant cultures as measured by ELISA. Data are represented as the mean (s.e.m.). *P = 0.015, significantly different.

**Fig. 3:**
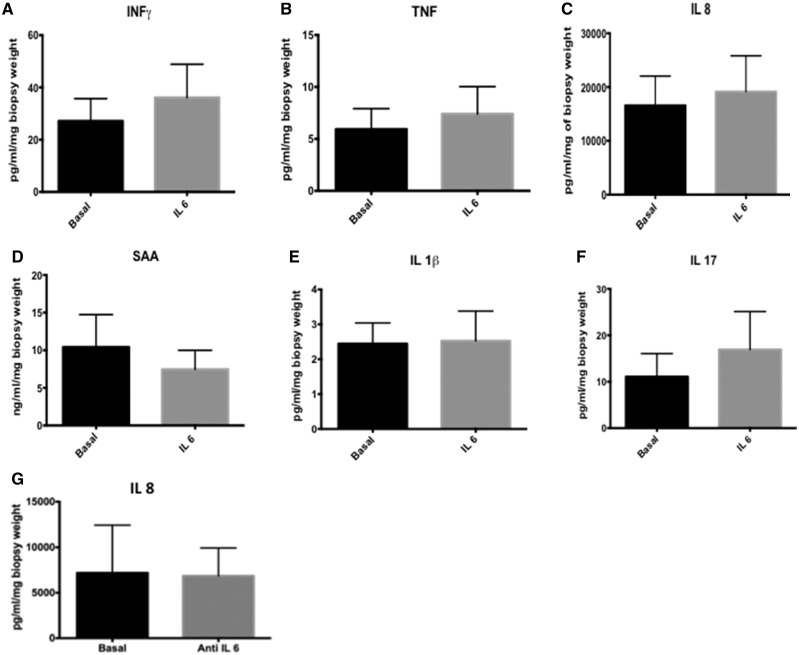
Stimulation of temporal artery explants with IL-6 (**A–F**) Stimulation of temporal artery explants (*n* = 28) from patients with GCA with IL-6 with or without soluble IL-6 receptor (sIL-6R) failed to induce expression of a number of key pro-inflammatory mediators known to contibute to the pathogenesis GCA. Data are represented as the mean (s.e.m.). (**G**) Treatment of the temporal artery explants with an IL-6 inhibitor did not block basal cytokine (IL-8) release.

In PBMC cultures established from patients with GCA, rhIL-6 (20 ng/ml) induced expression of IL-8 significantly, from basal levels of 386 ± 231.6 pg/ml to 678.1 ± 397.8 pg/ml (*P*=0.015; Fig. 2A). IL-6 induced VEGF levels from GCA temporal artery explants from basal levels of 7.74 ± 3.45 pg/ml to 107.3 ± 78.4 pg/ml (*P*=0.062; Fig. 2B), but no effect was observed for IFN-γ, TNF-α, IL-8, A-SAA, IL-1β and IL-17 (Fig. 3A–F). Differences were not observed in the presence or absence of the sIL6R, nor between those with a positive or negative temporal artery biopsy.

When temporal artery explants were cultured with anti-IL-6 *vs* IgG control, IL-6 inhibition failed to block basal IL-8 release (Fig. 3G).

The lack of effect of IL-6 on the temporal artery explants contrasted sharply with the dose-dependent increase in IL-8 expression observed after stimulation of RA synovial explants with rhIL-6 (Supplementary data, Figure S1).

### IL-6 does not promote cytoskeletal rearrangement or cell migration

**Fig. 4:**
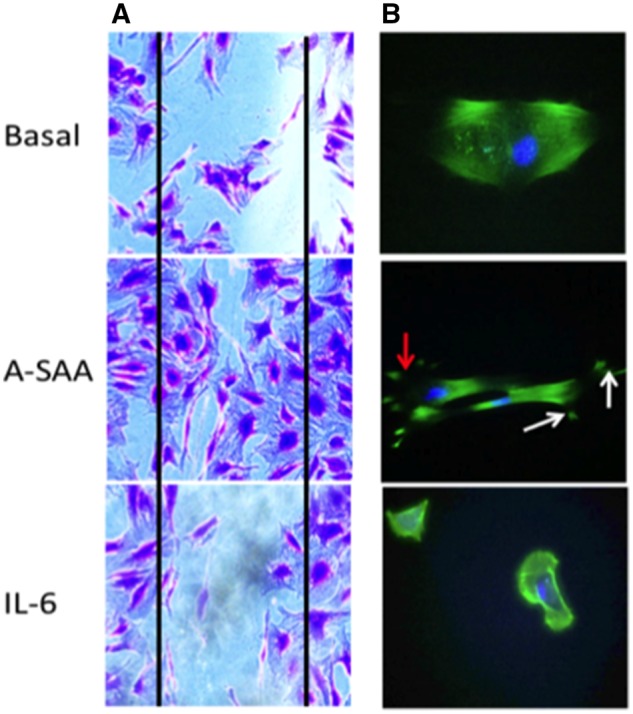
IL-6 does not promote myofibroblast migration nor cytoskeleton rearrangement (**A**) Representative micrography in basal and stimulated conditions, showing myofibroblasts repopulating the wound in response to A-SAA but clear wound margins still present after exposure to IL-6. (**B**) In basal and IL-6-stimulated conditions, intact F-actin fibres are present. However, after treatment with A-SAA, filiopodia formation (white arrows) and membrane ruffling (red arrow) are evident.

To examine the effects of IL-6 on cytoskeletal architecture, cultured myofibroblasts were treated with IL-6 (20 ng/ml) [and A-SAA (1 μg/ml) as a positive control] and immunostained for F-actin. Fig. 4B shows representative images of F-actin cytoskeleton disassembly and filopodial protrusion induced by A-SAA compared with intact actin fibres observed in the basal control. IL-6 had no effect on cytoskeletal rearrangement. To assess the effects of IL-6 on cell migration, wound repair assays were performed. A wound was created through the middle of each well, and cells were cultured with IL-6 (20 ng/ml) or A-SAA (1 μg/ml) for 24 h. Migration across the wound margin and repopulation by myofibroblasts was assessed. The duration of steroid treatment in advance of establishment of the explant culture did not affect myofibroblast outgrowths. Fig. 4A shows a clear wound in basal and IL-6-stimulated conditions, with minimal migration of cells across the wound margin, in contrast to A-SAA, which induced cell migration across the wound margins, resulting in almost complete closure of the wound.

### Effect of sIL-6R on signal transduction

**Fig. 5:**
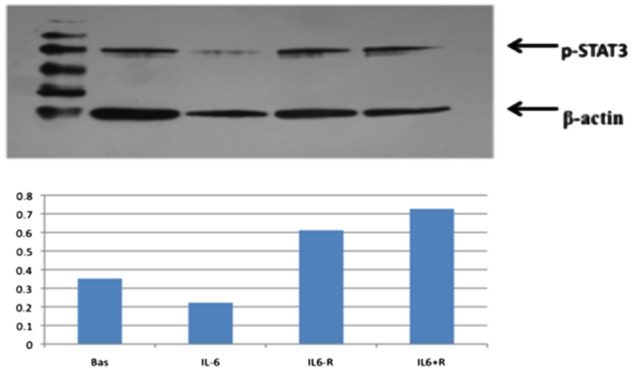
pSTAT3 expression Increased expression of pSTAT3 in response to stimulation with both IL-6 and the soluble IL-6 receptor (sIL-6R) but not IL-6 in isolation suggests that signalling has been activated but that sIL-6R is required to induce signal transduction in the temporal artery explants.

Western blot analysis revealed increased expression of pSTAT3 in response to the combination of IL-6 and sIL-6R, but not IL-6 alone, suggesting that the addition of the sIL-6R is necessary to induce signal transduction (Fig. 5).

## Discussion

In this study, we have demonstrated increased circulating IL-6 and sIL-6R in patients with GCA, with a subsequent decline in IL-6, but not sIL-6R, after glucocorticoid treatment. IL-6 stimulation of PBMCs from patients with GCA led to an increase in IL-8 production, whereas IL-6 stimulation of temporal artery explants failed to induce upregulation of any of the pro-inflammatory mediators assayed. In contrast, in the same concentrations, IL-6 activated pSTAT3 and increased VEGF production, in keeping with the known ability of IL-6 to induce angiogenesis. In contrast to A-SAA, IL-6 had no effect on cytoskeletal rearrangement or cell migration, which are processes felt to be relevant to the myointimal proliferation and luminal occlusion that leads to cranial ischaemic complications in GCA. These findings suggest that although IL-6 might play a key role in promoting systemic inflammation and the acute phase response, it might not be a key player in driving vascular inflammation in GCA.

In GCA, Th-1 and Th-17 lymphocytes have been identified as the predominant T-cell subsets orchestrating local and systemic inflammation [20, 21]. These separate T-cell lineages are associated with distinct cytokine profiles. IL-6 is one of the key Th-17-polarizing cytokines, with the subsequent IL-17 response thought to contribute to systemic inflammation, which is steroid sensitive, with a decrease in Th-17 cells evident post-treatment [22, 23]. IFN-γ is the signature cytokine of the Th-1 axis, and elevated IFN-γ levels are typically seen in patients with ischaemic events, suggesting that IFN-γ is crucial to the process driving vascular inflammation and, ultimately, luminal occlusion in GCA. Pro-inflammatory cytokine mRNA, including IL-6, has been identified from temporal artery specimens of patients with PMR; however, mRNA for IFN-γ has not been found, indicating that IFN-γ might have a role in the progression of subclinical vascular inflammation to overt arteritis [24].

We have found that circulating levels of IL-6 are elevated in active GCA and decrease post-treatment. However, considerable variability exists in pre-treatment levels of IL-6 and in the rate of decline after glucocorticoid treatment, consistent with previous studies [11, 12]. A number of studies have found persistently elevated IL-6 levels in patients in clinical remission, with a normal acute phase response [11, 12, 25]. These data, taken together, suggest that glucocorticoids transiently suppress IL-6 but do not correct the underlying mechanisms leading to its production. This is further supported by the finding that the short-term withdrawal of glucocorticoids results in the reappearance of significantly elevated levels of IL-6 in early and late disease [25]. Patients with higher IL-6 levels have been shown to have a more chronic relapsing disease course, with higher glucocorticoid requirements but without a clinically significant increase in disease or treatment-related adverse events [14].

Interestingly, higher IL-6 levels, which are correlated with the intensity of the systemic inflammatory response, have been found to be associated with a lower risk of cranial ischaemic events in GCA [26]. Although only two patients in our study had a cranial ischaemic complication, these patients had lower circulating IL-6 and lower spontaneous release of IL-6 from temporal artery explant cultures compared with those patients without a cranial ischaemic complication.

Increased circulating levels of sIL-6R have been reported in numerous inflammatory conditions. In PMR, elevated sIL-6R levels at baseline have been reported to be correlated with the number of relapses. In addition to its agonistic effects with IL-6, the sIL-6R is also thought to promote the half-life of IL-6, promote leucocyte recruitment, with consequent production of pro-inflammatory cytokines and proteases, and play a role in cellular proliferation and differentiation. However, in GCA, as in most disease processes, the precise pathological role of elevated sIL-6R remains unclear.

A number of recent case reports, case series, two open-label studies and one phase II, randomized, double-blind, placebo-controlled trial, in addition to GiACTA, a phase III, randomized double-blind, placebo-controlled trial designed to assess the efficacy and safety of tocilizumab in the treatment of GCA, have described the effects of tocilizumab, an IL-6R antagonist, in the treatment of GCA, with improvements reported in clinical symptoms and acute phase reactants, with significant CS-sparing effects [27–35]. Therefore, tocilizumab might provide tangible benefits for GCA patients in terms of improved quality of life, with reduction of disease-related symptoms and, potentially, a reduction in their significant burden of CS-related toxicity. A potential concern is that effective normalization of acute phase reactants and elimination of constitutional and PMR symptoms might mask the progression of large vessel vasculitis. However, the outcome measures in GiACTA do not include serial vascular imaging studies, thereby limiting assessment of persistence or progression of vascular inflammation and large vessel complications of GCA after tocilizumab treatment.

Previous studies in GCA have demonstrated that vascular inflammation can persist in many GCA patients despite CS therapy [23, 36], and large vessel complications can arise in the absence of clinical symptoms and with a normal acute phase response [37]. Although data are limited, a number of reports have suggested that tocilizumab might not treat large vessel vasculitis effectively in some patients with GCA [29, 31, 34, 38, 39].

Expression of IL-6 and TNF-α in GCA share a number of similarities. Patients with active GCA have elevated circulating levels of TNF-α and increased local expression in the temporal artery wall, with elevated levels of TNF-α correlating with the intensity of the systemic inflammatory response in GCA [13, 15, 40]. A number of case reports have also described benefits of infliximab and adalimumab in the treatment of CS-resistant GCA [41–43]; however, three randomized, double-blind, placebo-controlled trials of TNF-α inhibition failed to demonstrate a benefit [44–46]. These findings serve as a reminder that increased cytokine expression might reflect downstream effects and not necessarily have significant functional pathological relevance. In relationship to IL-6 biology, its effects are context specific; therefore, although IL-6 has been shown to be upregulated in AS, IBD and psoriasis, IL-6 inhibition in these disease states is not clinically effective [47, 48], further highlighting the importance of a greater understanding of the specific role of IL-6 in various disease processes.

The main limitation of the present study is the absence of a robust control. However, previously published work using this culture model has demonstrated a clear effect of serum amyloid A and Toll-like receptor 2 in inducing a pro-inflammatory cytokine response plus proliferation and migration of myofibroblasts. Stimulation of control synovial explant cultures and PBMCs with IL-6 with or without sIL-6R induced IL-8, and western blot analysis demonstrated upregulation of pSTAT3, confirming functional signalling activation in the explant cultures.

In conclusion, using our previously described *ex vivo* temporal artery culture model, exogenous administration of rhIL-6 failed to induce expression of a number of key pro-inflammatory mediators central to the pathogenesis of GCA in temporal artery explants. This would suggest that IL-6, although central to driving the systemic inflammatory component of GCA, might not play a significant role in the pathogenesis of overt large vessel arteritis. Long-term follow-up of IL-6 inhibition in GCA patients, including serial imaging studies, will be required to ascertain whether this hypothesis is borne out in clinical practice.

*Funding*: No specific funding was received from any funding bodies in the public, commercial or not-for-profit sectors to carry out the work described in this manuscript.

*Disclosure statement*: The authors have declared no conflicts of interest.

## Supplementary Material

rkz011_Supplementary_Data
