# Supplementary material for: Interleukin-6 does not upregulate pro-inflammatory cytokine expression in an ex vivo model of giant cell arteritis
Source: Rheumatol Adv Pract. 2019 May 6;3(1):rkz011. doi: 10.1093/rap/rkz011 (PMC6649906; doi:10.1093/rap/rkz011)
Supplement: rkz011_Supplementary_Data [file rkz011_supplementary_data.docx]

**SUPPLEMENTARY MATERIAL**

**Supplementary Table S1** **Clinical, demographic and laboratory parameters of the GCA patients included in the current study.**

| **Clinical Demographics** |  |
| --- | --- |
| Age (years; mean +/- SEM) | 73 +/- 11 |
| Gender | 21 Female, 7 Male |
| Biopsy Status | 11 Positive, 17 Negative |
| Polymyalgia rheumatica | 8 (28.6%) |
| Cranial Ischaemic Complication | 2 (7.1%) |
| Large Vessel Vasculitis* | 6 (21.4%) |
| ESR (mm/hr; mean +/- SEM) | 54 +/- 30 |
| CRP (g/dL; mean +/- SEM)  Fibrinogen (g/L; mean +/- SEM) | 43 +/- 40  4.05 +/- 0.36 |

Upper limit of normal in our laboratory for ESR, CRP and Fibrinogen are 30 mm/hr, 5 mg/dL and 4 g/L respectively. * Large vessel vasculitis as determined by vascular imaging study (CT or MR angiography).

**Supplementary Figure S1:**

**

**
